# Oculomic stratification of COVID-19 patients’ intensive therapy unit admission status and mortality by retinal morphological findings

**DOI:** 10.1038/s41598-024-68543-z

**Published:** 2024-09-12

**Authors:** Ella Courtie, Matthew Taylor, Dominic Danks, Animesh Acharjee, Thomas Jackson, Ann Logan, Tonny Veenith, Richard J. Blanch

**Affiliations:** 1 Institute of Inflammation and Ageing, University of Birmingham, Birmingham, UK; 2 Department of Ophthalmology, Queen Elizabeth Hospital Birmingham, University Hospitals Birmingham NHS Foundation Trust, West Midlands, UK; 3 Surgical Reconstruction and Microbiology Research Centre, University Hospitals Birmingham NHS Foundation Trust, Birmingham, UK; 4 University Hospitals Birmingham NHS Foundation Trust, Birmingham, UK; 5 University of Birmingham, Birmingham, UK; 6 Birmingham Women’s and Children’s NHS Foundation Trust, Birmingham, UK; 7 Alan Turing Institute, The British Library, London, UK; 8 Institute of Cancer and Genomic Sciences, College of Medical and Dental Sciences, University of Birmingham, Birmingham, B15 2TT UK; 9 Institute of Translational Medicine, University Hospitals Birmingham NHS Foundation Trust, Birmingham, B15 2TT UK; 10 MRC Health Data Research UK (HDR) Midlands, Birmingham, UK; 11 Centre for Health Data Research, University of Birmingham, Birmingham, B15 2TT UK; 12 Axolotl Consulting Ltd., Worcestershire, Droitwich, UK; 13 Division of Biomedical Sciences, Warwick Medical School, University of Warwick, Coventry, UK; 14 Critical Care Unit, Queen Elizabeth Hospital Birmingham, University Hospitals Birmingham NHS Foundation Trust, Birmingham, UK; 15 Department of Trauma Sciences, University of Birmingham, Birmingham, UK; 16 Academic Department of Military Surgery and Trauma, Royal Centre for Defence Medicine, Birmingham, UK

**Keywords:** Predictive markers, Predictive markers

## Abstract

To investigate if retinal thickness has predictive utility in COVID-19 outcomes by evaluating the statistical association between retinal thickness using OCT and of COVID-19-related mortality. Secondary outcomes included associations between retinal thickness and length of stay (LoS) in hospital. In this retrospective cohort study, OCT scans from 230 COVID-19 patients admitted to the Intensive Care Unit (ITU) were compared with age and gender-matched patients with pneumonia from before March 2020. Total retinal, GCL + IPL, and RNFL thicknesses were recorded, and analysed with systemic measures collected at the time of admission and mortality outcomes, using linear regression models, Pearson’s R correlation, and Principal Component Analysis. Retinal thickness was significantly associated with all-time mortality on follow up in the COVID-19 group (p = 0.015), but not 28-day mortality (p = 0.151). Retinal and GCL + IPL layer thicknesses were both significantly associated with LoS in hospital for COVID-19 patients (p = 0.006 for both), but not for patients with pneumonia (p = 0.706 and 0.989 respectively). RNFL thickness was not associated with LoS in either group (COVID-19 p = 0.097, pneumonia p = 0.692). Retinal thickness associated with LoS in hospital and long-term mortality in COVID-19 patients, suggesting that retinal structure could be a surrogate marker for frailty and predictor of disease severity in this group of patients, but not in patients with pneumonia from other causes.

## Introduction

The novel coronavirus COVID-19 (SARS-CoV-2) causes respiratory failure, with 4.9 million deaths globally, and severely affected patients requiring intensive treatment unit (ITU) support^1^. Multiorgan dysfunction and an exaggerated inflammatory response are associated with poor prognosis and mortality after COVID-19^2–4^.

Critically ill patients with COVID-19 and multiorgan dysfunction have an extensive injury to vascular endothelium with microcirculatory thrombus, manifesting as reduced functional capillary density^5^ and a functional reduction in oxygen delivery, propagating organ dysfunction^6^.

The retinal structure and microvasculature can be sequentially monitored non-invasively using optical coherence tomography (OCT). This laser imaging modality generates high-resolution cross-sectional retinal images, including of the macula and retinal nerve fibre layer (RNFL)^7,8^. Retinal neuronal and microvascular changes mirror systemic and cerebral pathology in health and diseases such as Parkinson’s and Alzheimer’s disease^6,9–11^. As retinal and cerebral circulations share similar mechanisms of autoregulation^12^, the retina may act as a biomarker for cerebral perfusion^8,13^.

A previous study involving 12 symptomatic COVID-19 patients used OCT to suggest retinal microvascular findings may be associated with COVID-19 neurological events, with results showing cotton wool spots, microhaemorrhages, and hyper-reflective lesions at the ganglion cell and inner plexiform layers in the participants^14^. However, this study did not detail the presence of pre-existing medical conditions, and the interpretation of the results was disputed^15^. A larger study involving 108 COVID-19 positive patients showed one in nine had retinal microvascular signs on colour fundus imaging and OCT, including microhaemorrhages, retinal vascular tortuosity, cotton wool spots, and hyper-reflectivity, which could be related to underlying cardiovascular and thrombotic alterations associated with COVID-19^3^.

The ability to image the retina non-invasively to detect ocular biomarkers of systemic disease is termed “oculomics”, which could predict disease progression by retinal microvascular and structural changes reflecting systemic change^16^. This study aimed to investigate retinal degenerative changes, as a potential biomarker for frailty, to predict health outcomes, including mortality after COVID-19. Thicknesses of the retinal ganglion cell layer (GCL) and RNFL—the neuronal layers most closely associated with cerebral structure and most sensitive to retinal ischaemia—were analysed to determine whether alterations in the retinal layer thickness correlated to outcomes of COVID-19 assessed by length of stay (LoS) in hospital and mortality after admission.

## Methods

### Study design and setting

This was a retrospective cross-sectional study investigating predictors of outcome after COVID-19 by retinal thickness analysis of previously acquired OCT images. This work uses data provided by patients and collected by the National Health Service (NHS) as part of their care and support at University Hospitals Birmingham NHS Foundation Trust and was approved by UHB Trust governance institutional review and permissions through the DeCOVID under application number [UHB-COV166]. This was entirely hospital electronic patient record-based, analysing results from the Queen Elizabeth Hospital Birmingham (QEHB) only, therefore no patient interaction was required. As only retrospective data were accessed, the determination by the DeCOVID ethics and institutional review process was that patient consent was not required. The study was conducted between December 2020 and August 2022, and we complied with the Declaration of Helsinki and the Data Protection Act 2018.

Key eligibility criteria were: (1) patients admitted to either the QEHB wards or ITU who tested COVID-19 positive and had at least one of the following investigations in the QEHB Ophthalmology Department within the past 10 years: “Fast Macula”, “Posterior Pole” (p-pole) or “RNFL” OCT scans and; (2) patients diagnosed with pneumonia from other causes before March 2020 who had the same OCT investigations in the QEHB Ophthalmology Department between 2010–2020 to act as age and gender-matched controls.

### Ophthalmic imaging

OCT Fast Macula (25 B-scans over an area of 5.7 mm^2^ at an automatic real time (ART) setting of 9 A-scans, averaged) and p-pole (61 B-scans over an area of 8.9 mm × 7.4 mm at an ART of 9) scans were exported from the Heidelberg EyeExplorer program (Heidelberg Engineering, Heidelberg, Germany). All scans had been obtained using the SPECTRALIS^®^ Heidelberg OCT2 table-top module (Heidelberg Engineering, Heidelberg, Germany).

### Health measures obtained

The following underlying diagnoses and health measures were collected for each patient included in the study: diabetes, ischaemic heart disease, chronic renal failure, chronic obstructive pulmonary disorder (COPD), National Early Warning Score (NEWS), glucose, blood pressure, C-reactive protein (CRP) levels, haematocrit, haemoglobin, eosinophil count, while blood cell (WBC) count, neutrophils, lymphocytes, albumin, estimated glomerular filtration rate (eGFR), and time to mortality following admission. Patient LoS in hospital is the number of days that an in-patient remains in hospital during a single admission event^17^. All measures were retrieved from the hospital database.

### OCT thickness extraction

The Fast Macula and p-pole scans were exported from Heidelberg EyeExplorer as E2E files for each patient and anonymised. The E2E files were converted to .VOL files using a research license and run through OCTseg—a custom made code—which automatically segmented and documented the total retinal thickness, combined GCL-plus-inner plexiform layer (GCL + IPL) thickness, and RNFL thickness^18^. Outliers with retinal thickness values more than 3 standard deviations from the mean were excluded.

### OCT thickness analysis

Patients were grouped by eye diagnosis (before thickness analysis was completed. Diagnoses included neovascular/dry/wet age-related macular degeneration, diabetic retinopathy, primary open angle glaucoma, diabetic maculopathy, and macula oedema.

### Statistics

We used univariate t test with false discovery rate correction (Benjamini-Hochberg, BH Procedure). A significance label was considered as p < 0.05. Associations with length of stay in COVID-19 patients and pneumonia control patients were estimated using linear regression, with corrected p values and variation explained (R^2^) reported. Correlation between measures was calculated using Pearson’s R correlation. In addition, Principal Component Analysis (PCA) was performed to study the variation in both cohorts. For PCA, data were normalised (standardised) using auto scaling. All analyses were performed in R (v4.2.3; R Core Team 2023)^19^.

### Informed consent statement

Informed consent requirement was waived by the DeCOVID institutional ethics committee for this retrospective study.

## Results

**Figure 1:**
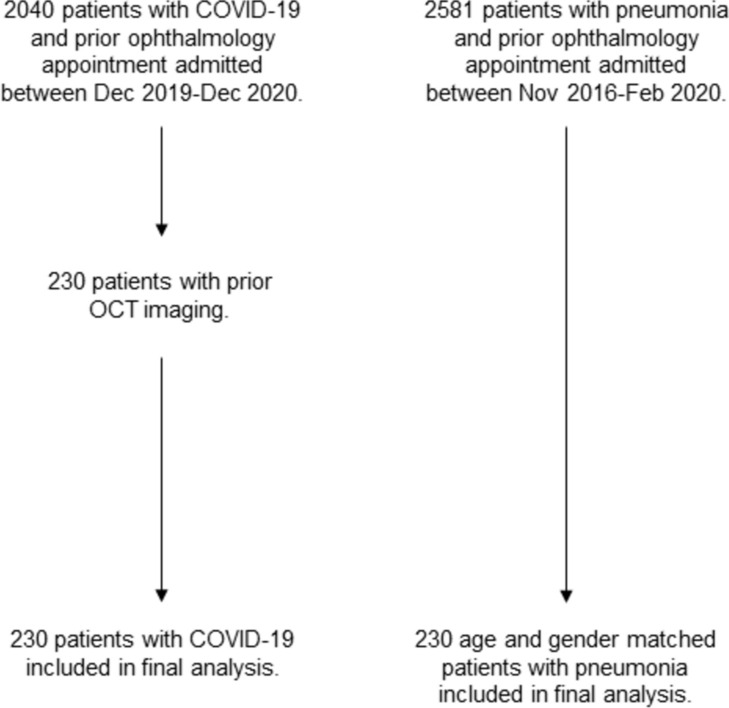
Study flowchart.

2040 COVID-19 positive patients were admitted to either the QEHB wards or ITU between December 2019 and December 2020, of which 230 patients had OCT imaging in the prior 10 years and were included in the final analysis. 2581 pneumonia patients who had attended a previous ophthalmology appointment were admitted to either the QEHB wards or ITU between November 2016 and February 2020 and from this group 230 age and gender-matched patients were included in the final analysis (all admitted before March 2020) (Fig. 1, Table 1). Mortality was assessed as “all time” at any point in follow up from the date of admission (0 to 1700 days, Table 1), as well as 28-day mortality after admission.

**Table 1:** Patient demographics table for COVID-19 and pneumonia groups.

Variable	Patients with COVID-19	Patients with pneumonia	P value
Male, N (%)	114	113	0.93
Female, N (%)	116	117	0.93
Age on admission (SD)	74.5 (16.5)	74.5 (16.4)	1.00
Comorbidities, N (%)			
Diabetes mellitus	86 (37.4)	74 (32.2)	0.28
Ischaemic heart disease	11 (4.8)	18 (7.8)	0.25
Chronic renal failure	41 (17.8)	19 (8.3)	0.03
COPD	22 (9.6%)	35 (15.2)	0.09
Eye disorders, N (%)			
Dry age-related macular degeneration	17 (7%)	18 (8%)	0.87
Diabetic macular oedema	13 (6%)	13 (6%)	1.00
Diabetic retinopathy	37 (16%)	20 (9%)	0.02
Glaucoma	24 (10%)	15 (7%)	0.15
None (no retinal diagnosis)	103 (45%)	131 (57%)	0.06
Other	23 (10%)	12 (5%)	0.06
Wet age-related macular degeneration	13 (6%)	21 (9%)	0.17
Mortality, N (%)	77 (23.3)	147 (35.5)	< 0.01
Time to mortality, days Range (mean)	0–143 (20.5)	0–1700 (489)	0.016
NEWS on admission (SD)	4.16 (3.3)	4.38 (3.0)	0.56
CRP peak, mg/L (SD)	139.2 (110.3)	162.1 (124.6)	0.04
LoS, days	11.8 (14.4)	11.2 (12.1)	0.63
HCT lowest (SD)	0.34 (0.1)	0.32 (0.1)	0.01
HGB lowest, g/L (SD)	111.9 (23.8)	107.2 (22.9)	0.03
WBC on admission, × 10^9^/L (SD)	8.36 (4.7)	12.6 (6.1)	< 0.001
Neutrophils on admission, × 10^9^/L (SD)	6.57 (4.4)	10.3 (5.8)	< 0.001
Neutrophils peak, × 10^9^/L (SD)	9.30 (6.2)	12.4 (6.6)	< 0.001
ALB lowest, g/L (SD)	26.9 (6.6)	31.9 (6.8)	< 0.001
EGFR lowest, ml/min/1.73 m^2^ (SD)	44.7 (23.9)	48.5 (22.7)	0.06

Demographics and clinical characteristics of the participants included in the study given as mean (standard deviation). P-values of < 0.05 are considered significant.

*N* number, *SD* standard deviation, *COPD* chronic obstructive pulmonary disorder, *CRP* C-reactive protein, *HCT* haematocrit, *HGB* haemoglobin, *WBC* white blood cells, *ALB* albumin, *EGFR* estimated glomerular filtration rate.

### LoS in hospital associated with retinal thickness in patients with COVID-19

Retinal and GCL + IPL layer thicknesses were both strongly associated with LoS in patients with COVID-19 (p = 0.006 for both; Table 2, Supplementary Fig. 1), but not in patients with pneumonia (p = 0.706 and 0.989 respectively; Table 2). RNFL thickness was weakly associated with LoS in hospital in the COVID-19 group (p = 0.097) but not in patients with pneumonia (p = 0.692).

**Table 2:** Associations with length of stay in patients with COVID-19 and patients with pneumonia.

Group	Measure	Mean ± SD	P-value	% Variation R^2^
COVID-19	**Retinal thickness**	**298.4 ± 28.13**	0.006*	**3.385**
	**GCL + IPL thickness**	**61.39 ± 13.93**	0.006*	**3.469**
	RNFL thickness	47.79 ± 13.34	0.097	1.267
	**Albumin lowest**	**26.85 ± 6.609**	< 0.001*	**21.45**
	**Neutrophils peak**	**9.299 ± 6.175**	< 0.001*	**19.96**
	**Haemoglobin lowest**	**111.9 ± 23.81**	< 0.001*	**16.37**
	**Haematocrit lowest**	**0.340 ± 0.070**	< 0.001*	**16.24**
	**CRP peak**	**139.2 ± 110.3**	< 0.001*	**9.961**
	**WBC on admission**	**8.356 ± 4.661**	0.001*	**4.670**
	**Neutrophils on admission**	**6.573 ± 4.375**	0.004*	**3.800**
	**EGFR lowest**	**44.67 ± 23.87**	0.008*	**3.229**
	Age on admission	74.47 ± 16.48	0.113	1.151
Pneumonia	Retinal thickness	295.4 ± 19.34	0.706	0.153
	GCL + IPL thickness	56.92 ± 12.06	0.989	0.000
	RNFL thickness	47.84 ± 12.74	0.692	0.168
	**Albumin lowest**	**32.61 ± 6.718**	0.003*	**8.916**
	**Neutrophils peak**	**12.36 ± 7.111**	0.003*	**8.842**
	**Haemoglobin lowest**	**106.5 ± 22.32**	< 0.001*	**17.10**
	**Haematocrit lowest**	**0.320 ± 0.067**	< 0.001*	**16.68**
	CRP peak	154.7 ± 114.7	0.114	2.639
	WBC on admission	12.18 ± 5.789	0.655	0.213
	Neutrophils on admission	9.995 ± 5.460	0.659	0.208
	**EGFR lowest**	**47.46 ± 22.66**	0.040*	**4.412**
	Age on admission	75.78 ± 14.71	0.989	0.000

Rows in bold text represent statistically significant differences in measures, where p ≤ 0.05.

*SD* standard deviation, *GCL* ganglion cell layer, *IPL* inner plexiform layer, *RNFL* retinal nerve fibre layer, *CRP* C-reactive protein, *WBC* white blood cells, *EGFR* estimated glomerular filtration rate. * p < 0.05.

### LoS associated with acute inflammatory blood markers in patients with COVID-19

The LoS in hospital for patients with COVID-19 was significantly associated with blood inflammatory markers, with the strongest association for levels of peak neutrophils, WBC, and CRP levels (p < 0.001 for all measures) (Table 2, Supplementary Fig. 1), while only peak neutrophil levels were significantly associated with LoS for patients with pneumonia but not WBC or CRP levels (p = 0.003, 0.655, 0.144 respectively; Supplementary Fig. 2). NEWS was not significantly different between patients with pneumonia and patients with COVID-19 (4.38 and 4.16 respectively, p = 0.56; Table 1), suggesting similar levels of disease severity. LoS in hospital was also significantly associated with low haemoglobin and haematocrit both in patients with COVID-19 (p < 0.001) and patients with pneumonia (p < 0.001; Table 2).

### Retinal thickness associated with all-time mortality in patients with COVID-19 and pneumonia, but not 28-day mortality

**Figure 2:**
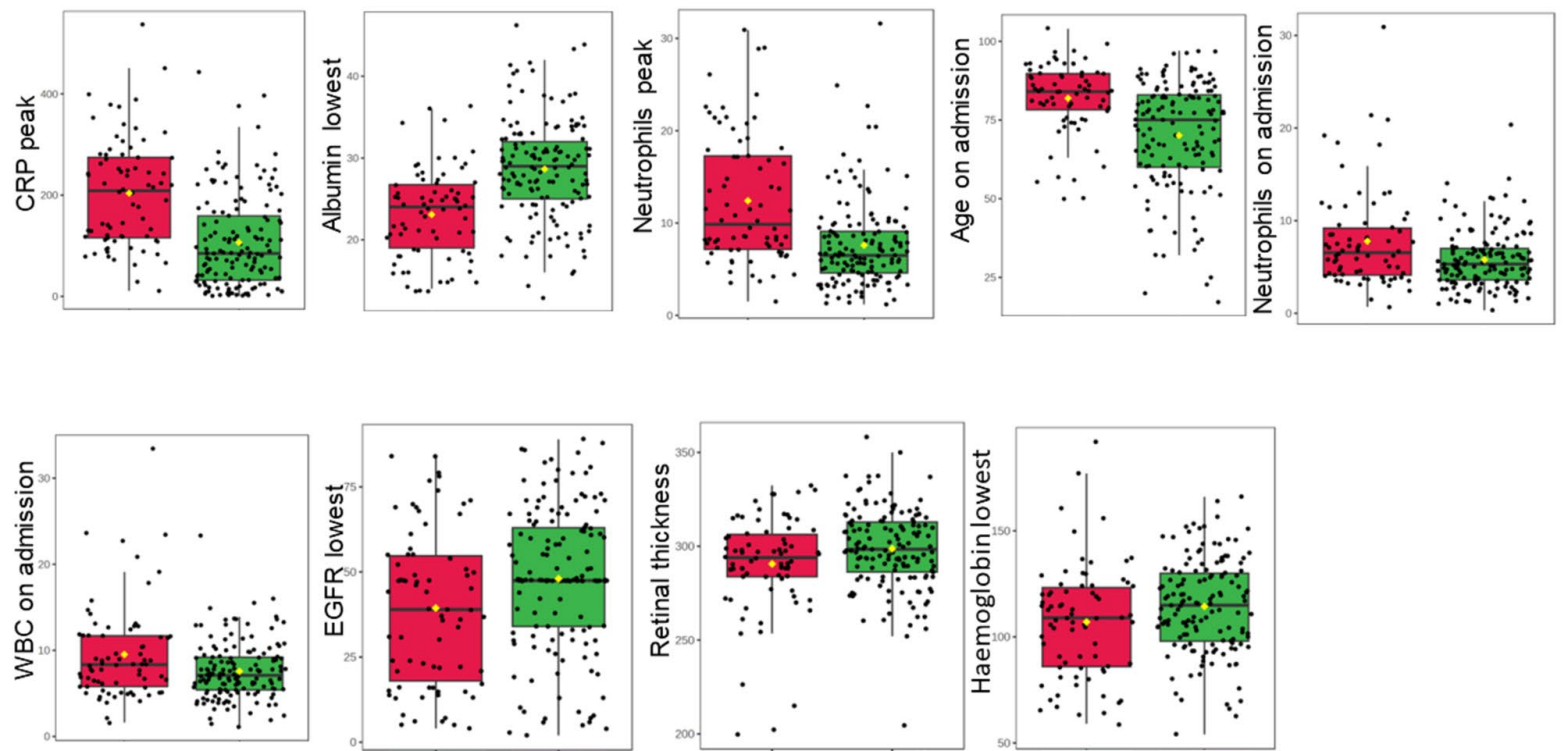
Boxplots of significant associations with all time mortality in patients with COVID-19. The green boxplots represent patients who survived, the red boxplots represent patients who died. Each black dot represents a patient with COVID-19. The yellow rhombus represents the mean value of the distribution. *CRP* C-reactive protein, *WBC* white blood cells, *EGFR* estimated glomerular filtration rate.

**Figure 3:**
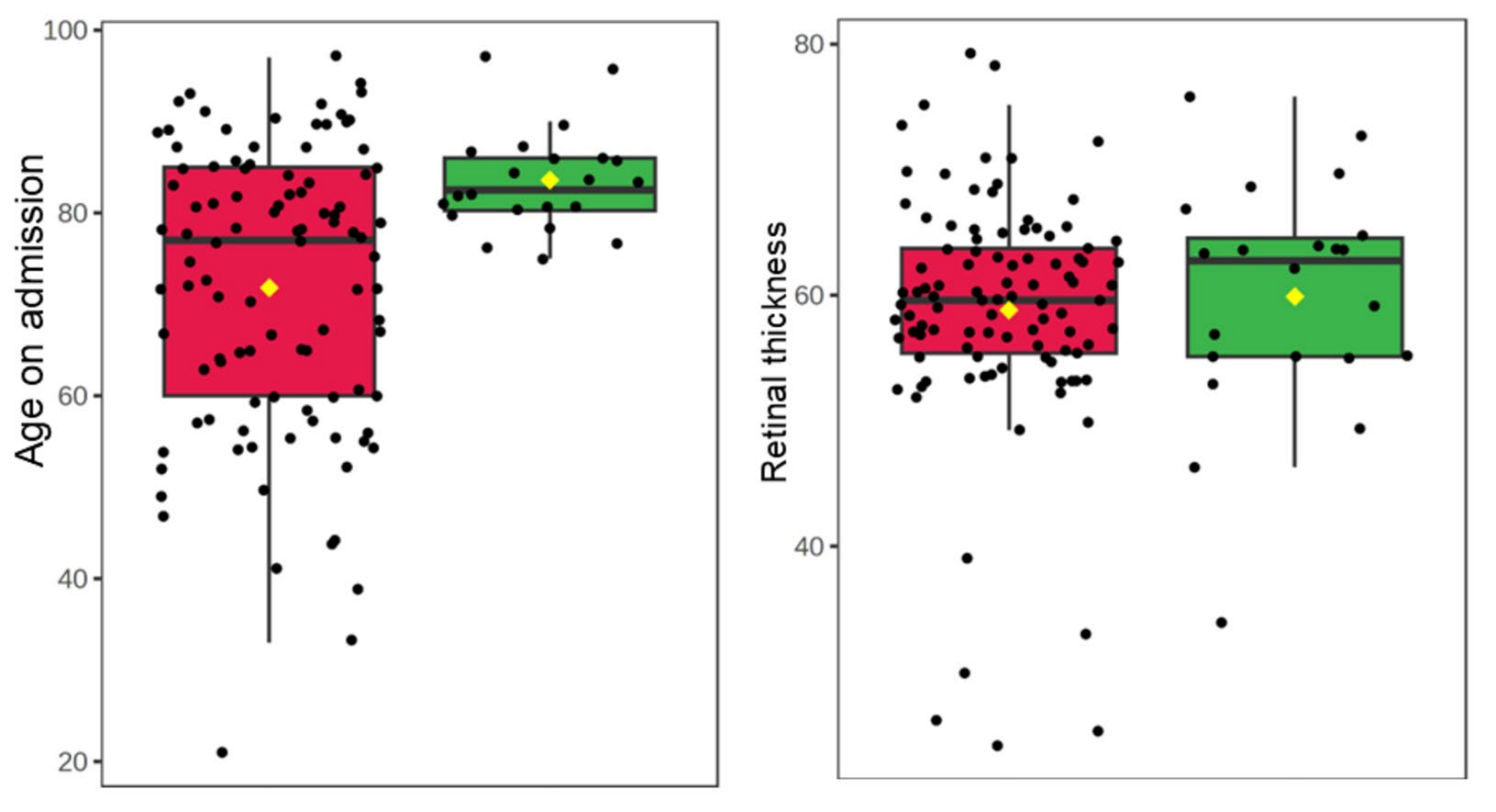
Boxplots to show associations with all time mortality of patients with pneumonia and significant markers. The green boxplots represent patients who survived, the red boxplots represent patients who died. Each black dot represents a patient with pneumonia. The yellow rhombus represents the mean value of the distribution.

Most blood markers were significantly associated with all-time mortality in COVID-19 patients, with the strongest association with peak CRP level, CRP on admission, and lowest albumin level (p < 0.001 for all; Fig. 2, Table 3). Total retinal thickness was also associated with all-time mortality (p = 0.015), although GCL + IPL and RNFL thickness were not (Fig. 2, Table 3). No blood markers were significantly associated with all-time mortality in pneumonia patients (Fig. 3, Table 3), but similar to the COVID-19 group, retinal thickness was associated with all-time mortality in pneumonia patients (p = 0.024; Fig. 3, Table 3).

**Table 3:** Associations with all-time mortality.

Group	Measure	P-value	FDR
COVID-19	**Retinal thickness**	0.015*	**0.033**
	GCL + IPL thickness	0.694	0.671
	RNFL thickness	0.452	0.502
	**Albumin lowest**	< 0.001*	** < 0.001**
	**Neutrophils peak**	< 0.001*	** < 0.001**
	**Haemoglobin lowest**	0.032*	**0.065**
	Haematocrit lowest	0.065	0.114
	**CRP peak**	< 0.001*	** < 0.001**
	**WBC on admission**	0.001*	**0.004**
	**Neutrophils on admission**	0.001*	**0.002**
	**EGFR lowest**	0.006*	**0.013**
	**Age on admission**	< 0.001*	** < 0.001**
Pneumonia	**Retinal thickness**	0.007*	**0.094**
	GCL + IPL thickness	0.175	0.474
	RNFL thickness	0.981	0.993
	Albumin lowest	0.710	0.959
	Neutrophils peak	0.323	0.726
	Haemoglobin lowest	0.082	0.345
	Haematocrit lowest	0.170	0.474
	CRP peak	0.681	0.959
	WBC on admission	0.952	0.993
	Neutrophils on admission	0.883	0.993
	EGFR lowest	0.054	0.345
	**Age on admission**	< 0.001*	**0.002**

Rows in bold text represent statistically significant differences in measures, where p ≤ 0.05.

*FDR* false discovery rate, *GCL* ganglion cell layer, *IPL* inner plexiform layer, *RNFL* retinal nerve fibre layer, *CRP* C-reactive protein, *WBC* white blood cells, *EGFR* estimated glomerular filtration rate.

**Figure 4:**
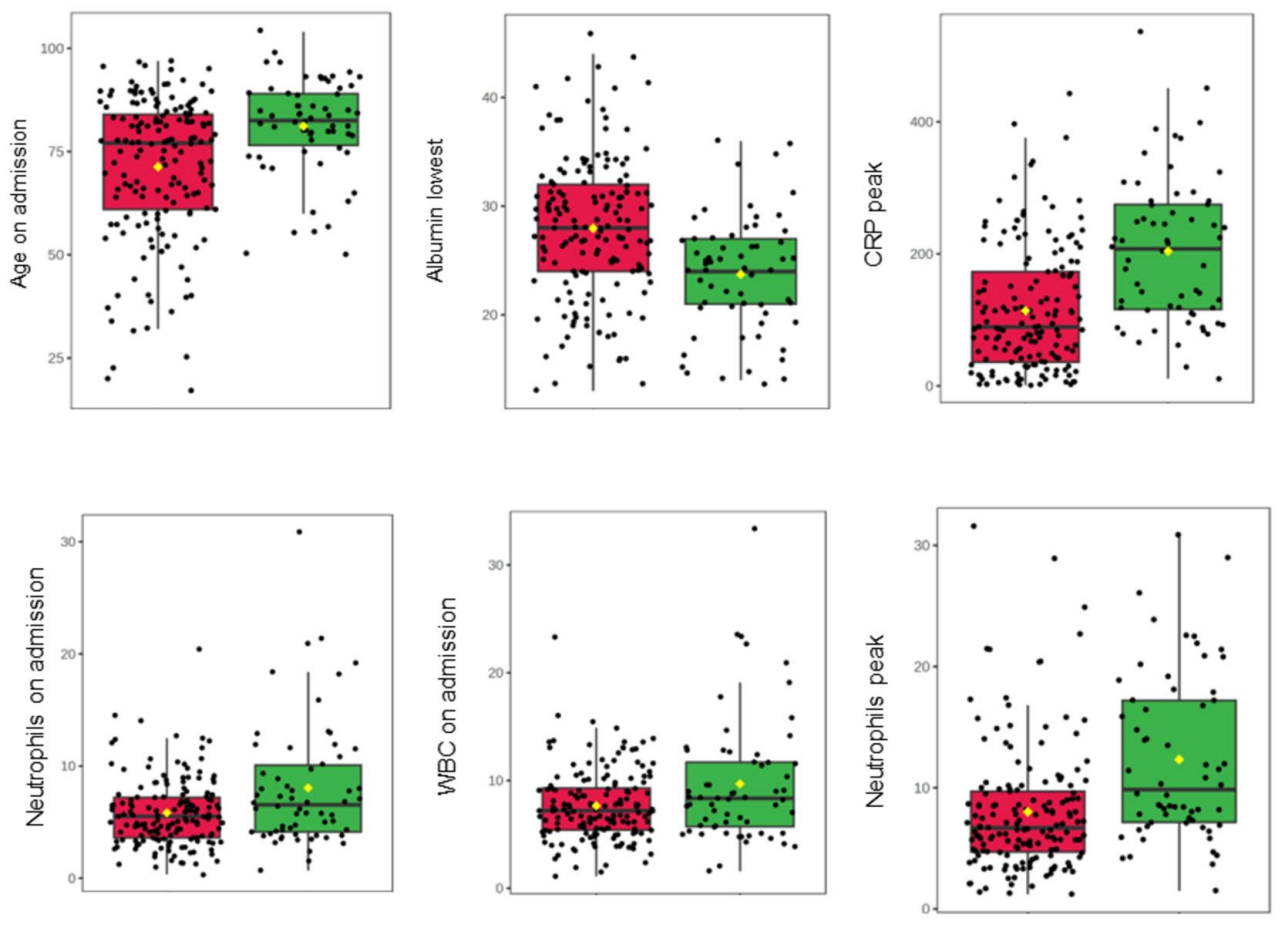
Boxplots to show associations with 28-day mortality of patients with COVID-19 and significant markers. The green boxplots represent patients who survived, the red boxplots represent patients who died. Each black dot represents a patient with COVID-19. The yellow rhombus represents the mean value of the distribution. *CRP* C-reactive protein, *WBC* white blood cells.

**Figure 5:**
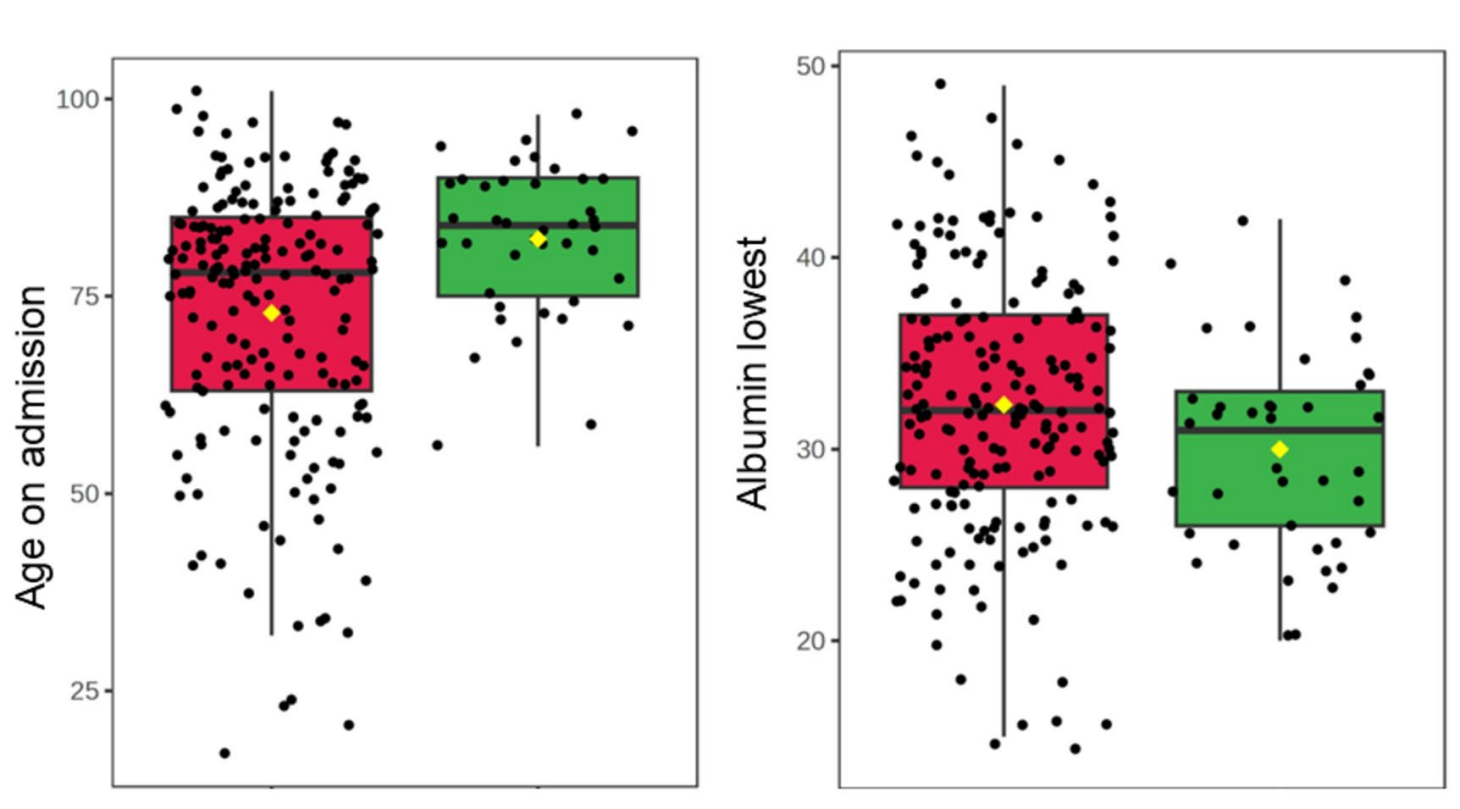
Boxplots to show associations with 28-day mortality of patients with pneumonia and significant markers. The green boxplots represent patients who survived, the red boxplots represent patients who died. Each black dot represents a patient with pneumonia. The yellow rhombus represents the mean value of the distribution.

No retinal thickness measurements were associated with 28 day mortality in patients with either COVID-19 (retinal thickness p = 0.151, GCL + IPL p = 0.610, RNFL p = 0.480) or pneumonia (GCL + IPL p = 0.344, RNFL p = 0.813) except for total retinal thickness in patients with pneumonia, which was weakly associated with 28 day mortality (p = 0.067; Fig. 4, Table 4). The blood markers most strongly associated with 28-day mortality in COVID-19 patients were lowest albumin level, peak neutrophil count, and peak CRP (p < 0.001), while the strongest markers for pneumonia patients were lowest albumin level and age on admission (p = 0.043 and p = 0.001 respectively, Fig. 5, Table 4).

**Table 4:** Associations with 28-day mortality.

Group	Measure	P-value	FDR
COVID-19	Retinal thickness	0.151	0.244
	GCL + IPL thickness	0.610	0.610
	RNFL thickness	0.480	0.533
	**Albumin lowest**	< 0.001*	** < 0.001**
	**Neutrophils peak**	< 0.001*	** < 0.001**
	Haemoglobin lowest	0.200	0.286
	Haematocrit lowest	0.328	0.410
	**CRP peak**	< 0.001*	** < 0.001**
	**WBC on admission**	0.001*	**0.004**
	**Neutrophils on admission**	** < 0.001**	**0.001**
	EGFR lowest	0.098	0.184
	**Age on admission**	< 0.001*	** < 0.001**
Pneumonia	Retinal thickness	0.067	0.347
	GCL + IPL thickness	0.344	0.813
	RNFL thickness	0.579	0.947
	**Albumin lowest**	**0.043**	**0.347**
	Neutrophils peak	0.207	0.617
	Haemoglobin lowest	0.561	0.947
	Haematocrit lowest	0.713	0.947
	CRP peak	0.624	0.947
	WBC on admission	0.618	0.947
	Neutrophils on admission	0.630	0.947
	EGFR lowest	0.060	0.347
	**Age on admission**	0.001*	**0.021**

Rows in bold text represent statistically significant differences in measures, where p ≤ 0.05.

*FDR* false discovery rate, *GCL* ganglion cell layer, *IPL* inner plexiform layer, *RNFL* retinal nerve fibre layer, *CRP* C-reactive protein, *WBC* white blood cells, *EGFR* estimated glomerular filtration rate.

### Associations with retinal layer thicknesses in retinal diagnostic subgroups

The retinal diagnoses for which the included patients attended the ophthalmology clinic are reported in Table 1, with comparable proportions of patients with diabetic macular oedema and wet age-related macular degeneration, but a higher proportion of patients with diabetic retinopathy and glaucoma in the COVID-19 group compared to the pneumonia group. Subgroup analysis of the association between LoS and GCL+ thickness in the different retinal diagnostic groups in patients with COVID-19 revealed the same directionality of association in the diagnostic groups: other (p = 0.04), glaucoma (p = 0.05), none (p = 0.11), dry age-related macular degeneration (p = 0.57), and diabetic macular oedema (p = 0.64); but not in: diabetic retinopathy (p = 0.92) or wet age-related macular degeneration (p = 0.83). Similarly, for the association between LoS and retinal thickness, subgroup analysis revealed the same directionality of association in the diagnostic groups: none (p < 0.001), glaucoma (p = 0.009), diabetic retinopathy (p = 0.85), and wet age-related macular degeneration (p = 0.43) but not the other groups. For the association with mortality in patients with COVID-19, retinal thickness retained the same (non-significant) association in all retinal diagnostic groups except diabetic macular oedema, whilst a model could not be fit for the same subgroup analysis in patients with pneumonia.

## Discussion

When analysing previously acquired structural OCT images from patients admitted to hospital with COVID-19, retinal thickness and GCL + IPL layer thickness were significantly associated with the LoS in hospital in this group of patients but not in patients with pneumonia, whilst retinal thickness was predictive of all-time but not 28 day mortality for patients with both COVID-19 and pneumonia. These findings suggest that retinal thickness could be a predictor of COVID-19 and pneumonia severity and potentially represent a marker for frailty, supporting wider associations between systemic disease and retinal signs^20–23^.

Frailty is a medical syndrome which increases an individual’s vulnerability to and risk of adverse health outcomes when exposed to a stressor^24^, which considers weight loss, exhaustion, low activity, slowness, and weakness in the screening process^24^. Frailty is an independent predictor of mortality in COVID-19 patients^25^, with an increased risk of mortality in frail patients who were under 65 years old as well as an increased incidence of ITU admission^26^. Frailty is a risk factor for increased susceptibility to and severity of pneumonia in adults ≥ 65 years old^27^. The severity of physical frailty assessed by the Fried phenotype (which includes physical inactivity, exhaustion, weakness, weight loss, and slow walking speed) correlated with elevated total white matter hyperintensity and lower grey matter volume^28^. The neuroretina contains central nervous system neurons and retinal perfusion and structural changes may reflect cerebral changes in illnesses from stroke to multiple sclerosis in addition to systemic diseases such as sepsis and cardiac disease^6,29^. An association between retinal changes and frailty could therefore reflect the manifestation of multiple neurodegenerative and cardiovascular diseases. We found that retinal thickness was associated with all-time mortality in both patients with COVID-19 and pneumonia, though not 28-day mortality, which could support the ability of retinal thickness to be a marker of long-term frailty, consistent with the stronger findings in patients with COVID-19 and LoS. We were unable to control for frailty, as frailty was only recorded routinely in the COVID-19 group.

Both retinal thickness and GCL + IPL thickness significantly associated with LoS in COVID-19 patients, but not in pneumonia patients. LoS is not just dependent on the medical reason for admission and can be affected by patient demographics, past medical history, treatment complexity, and complications^17^. Log regression statistics, machine learning, and data mining are all used to predict LoS with the aim to predict the level of care that may be required for the patient, although it remains a complicated area to model due to external factors^17^. As retinal thickness is associated with COVID-19 LoS, retinal assessments may be able to contribute to predictive markers for patient LoS in this group. However, retinal thickness was not associated with LoS in pneumonia patients, which may be because this group of patients was less severely ill (although NEWS was equal between the groups) or the fact that we were not able to separate community acquired and hospital acquired pneumonia, so the pneumonia patient group may be more heterogenous. During the pandemic, hospital admissions were under greater pressure than before, with the consequence that only the most severely ill and vulnerable patients were admitted^30^. The difference between COVID-19 and pneumonia patients could relate to the frailty of the patient prior to admission, with a stronger signal from retinal thickness in the frailer COVID-19 group but not in the less frail pneumonia group. Blood infection markers were also associated with LoS and all-time mortality, and therefore associate with illness severity, although these results came from blood collected during admission. Because retinal layer thicknesses showed associations with these outcomes, and the OCT images were taken before admission, retinal thickness may predict these outcomes and susceptibility to severe disease.

There was a higher number of patients with COVID-19 who had chronic renal failure compared to patients with pneumonia. A recent study found that patients with chronic kidney disease had retinal and choroidal thinning on OCT compared with healthy volunteers^31^, with similar results shown in late-stage chronic kidney disease in another study^32^. Renal failure also represents a risk for severe COVID-19, which may also cause acute kidney injury. It is therefore unsurprising that renal failure would be more common and EGFR lower in patients with COVID-19 than in patients with pneumonia. Many other systemic diseases may also manfest in retinal thickness or perfusion changes^6^. The PCA analysis examines the contribution of each included predictor independently, and it would be informative in future larger studies to examine the interaction between renal failure, retinal thickness and disease susceptibility.

The associations between retinal thickness, LoS and mortality were present across most diagnostic groups, although the study was most likely not powered for these subgroup analyses. In particular retinal thickness associated with LoS and mortality in patients with no retinal diagnosis, and glaucoma, but not in patients with age-related macular degeneration or diabetic maculopathy, which may reflect greater variability in the data caused by retinal oedema in these cohorts.

## Conclusions

The associations between retinal thickness and LoS in hospital in COVID-19 patients, and retinal thickness and all-time mortality in both patients with COVID-19 and those with pneumonia, suggests that retinal structure could be a marker of frailty and a predictor of disease severity in this group.

## Supplementary Information

Supplementary Figures.

## Data Availability

Data is provided within the manuscript or supplementary information files. The datasets used and/or analysed during the current study can be provided on reasonable request to Lt Col Richard Blanch (corresponding author) and subject to a data transfer agreement with UHB NHS Foundation Trust, which is the data owner.

## Abbreviations

COVID-19: Coronavirus disease, SARS-CoV-2
ITU: Intensive treatment unit
OCT: Optical coherence tomography
RNFL: Retinal nerve fibre layer
GCL: Ganglion cell layer
LoS: Length of stay
NHS: National health service
QEHB: Queen Elizabeth Hospital Birmingham
p-pole: Posterior pole
ART: Automatic real time
COPD: Chronic obstructive pulmonary disorder
NEWS: National Early Warning Score
CRP: C-reactive protein
WBC: White blood cell
eGFR: Estimated glomerular filtration rate
GCL + IPL: Ganglion cell layer plus inner plexiform layer
PCA: Principle component analysis
FDR: False discovery rate
