# Supplementary material for: Oculomic stratification of COVID-19 patients’ intensive therapy unit admission status and mortality by retinal morphological findings
Source: Sci Rep. 2024 Sep 12;14:21312. doi: 10.1038/s41598-024-68543-z (PMC11393335; doi:10.1038/s41598-024-68543-z)
Supplement: Supplementary file 1 — Supplementary Figures. [file 41598_2024_68543_MOESM1_ESM.docx]

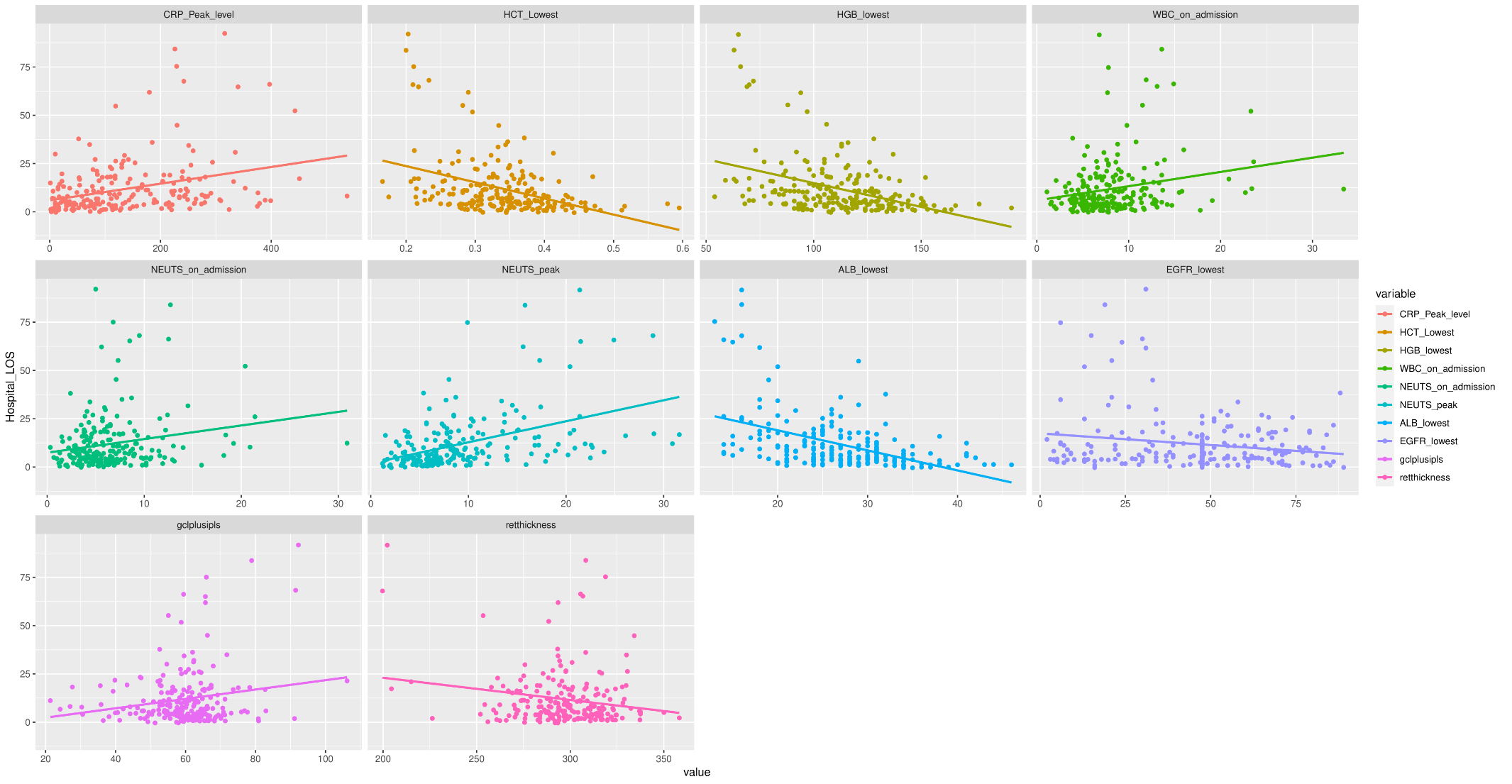


**Supplementary Figure 1. Scatter plots to show associations with LOS in hospital of patients with COVID-19 and significant markers.** Hospital LOS is in days and represented by the y-axis, while the marker described in the graph title represents the x-axis. Variables are denoted in the key. Abbreviations: LOS: length of stay in hospital; CRP: C-reactive protein; HCT: haematocrit; HGB: haemoglobin; WBC: white blood cells; NEUTS: neutrophils; ALB: albumin; EGFR: estimated glomerular filtration rate; GCLplusIPL: ganglion cell layer plus inner plexiform layer; retthickness: retinal thickness.


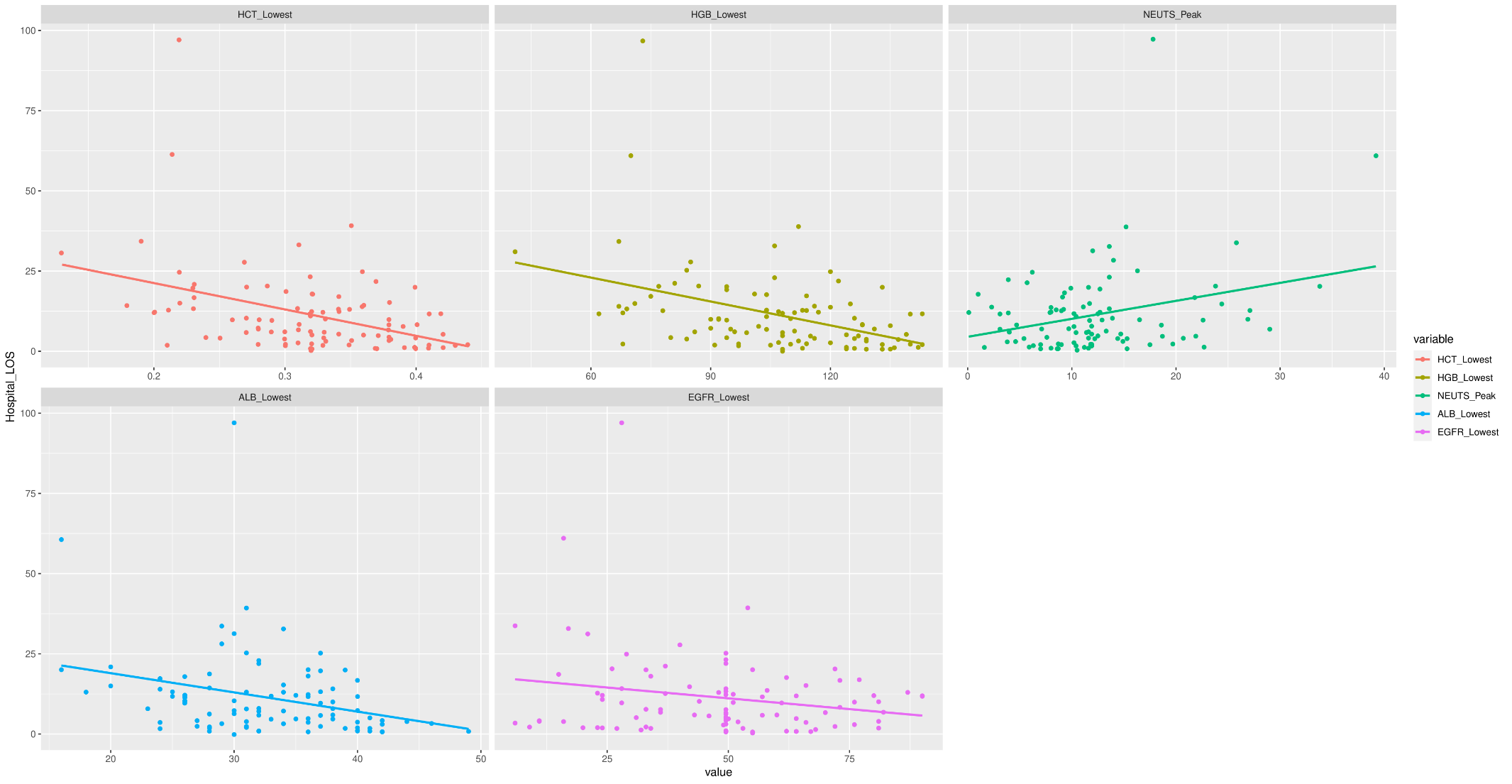


Supplementary Figure 2. **Scatter plots to show associations with LOS in hospital of patients with pneumonia and significant markers.** Hospital LOS is in days and represented by the y-axis, while the marker described in the graph title represents the x-axis. Variables are denoted in the key. Abbreviations: LOS: length of stay in hospital; HCT: haematocrit; HGB: haemoglobin; NEUTS: neutrophils; ALB: albumin; EGFR: estimated glomerular filtration rate.
